# Formalization of taxon-based constraints to detect inconsistencies in annotation and ontology development

**DOI:** 10.1186/1471-2105-11-530

**Published:** 2010-10-25

**Authors:** Jennifer I Deegan (née Clark), Emily C Dimmer, Christopher J Mungall

**Affiliations:** 1 European Bioinformatics Institute, Wellcome Trust Genome Campus, Hinxton, Cambridge, CB10 1SD, UK; 2 240C Building 64, Lawrence Berkeley National Lab, 1 Cyclotron Road, Berkeley CA 94720

## Abstract

**Background:**

The Gene Ontology project supports categorization of gene products according to their location of action, the molecular functions that they carry out, and the processes that they are involved in. Although the ontologies are intentionally developed to be taxon neutral, and to cover all species, there are inherent taxon specificities in some branches. For example, the process 'lactation' is specific to mammals and the location 'mitochondrion' is specific to eukaryotes. The lack of an explicit formalization of these constraints can lead to errors and inconsistencies in automated and manual annotation.

**Results:**

We have formalized the taxonomic constraints implicit in some GO classes, and specified these at various levels in the ontology. We have also developed an inference system that can be used to check for violations of these constraints in annotations. Using the constraints in conjunction with the inference system, we have detected and removed errors in annotations and improved the structure of the ontology.

**Conclusions:**

Detection of inconsistencies in taxon-specificity enables gradual improvement of the ontologies, the annotations, and the formalized constraints. This is progressively improving the quality of our data. The full system is available for download, and new constraints or proposed changes to constraints can be submitted online at https://sourceforge.net/tracker/?atid=605890&group_id=36855.

## Background

The Gene Ontology (GO) Project [1,2] provides ontologies for the categorization of gene products according to their locations of action, the molecular functions that they carry out, and the processes that they are normally involved in. These categorizations propagate up the ontology graph structure, from specific classes to more general classes. This is known as the "true path rule", and great care is taken in ontology development to ensure that the true path rule holds, and detection of errors is a high priority. Over 56 million GO annotations are currently available from the GO Consortium, supplying functional information for almost 220,000 different taxonomic groups (January 2010). Many of these GO annotations have been generated through manual curation, in which a curator extracts data from published literature. Others have been generated by reviewed computational predictions. A large number are also produced by minimally supervised automatic prediction pipelines. A number of different automated prediction methods are applied by members of the GO Consortium. These methods include transfer of manual GO annotations to closely related orthologs (Ensembl [3], GO reference genome project [4]), use of protein signatures to predict functionally-similar proteins (InterPro [5]), and mapping of external functional concepts to equivalent GO classes (UniProtKB [6]). The number and type of GO annotations available for a set of gene products relies heavily on the amount of funded curation work and the experimental literature available. Therefore, although many model organisms have a large amount of manual GO annotation, automated GO annotation is the principal source of functional data for many other organisms. Each GO annotation records the type of evidence associating a gene product with a particular GO class using an evidence code. Sixteen evidence codes are used to describe manual GO annotation efforts, and one code, 'IEA' (Inferred from Electronic Annotation), describes all automatically-predicted GO annotations http://www.geneontology.org/GO.evidence.shtml. There are many different methods of automatically predicting GO annotations, and one of the most popular is the InterPro2GO method [7], which uses a mapping file between protein domains and GO classes to predict annotations on the basis of domain predictions.

GO contains in excess of 28,000 classes, and the GO as a whole is intended to cover the full range of species. GO classes are defined to be taxon neutral, avoiding reliance on taxon information for full definition of the given process, function, or component. As an example of this, the class 'lactation'; GO:0007595 is defined as 'The secretion of milk by the mammary gland.' rather than 'The secretion of milk by the mammary gland in mammals.'. In classes such as this, however, there is obvious implicit taxon specificity, such that this class should only be used to categorize gene products from mammalian species. It is possible to automatically detect errors in the ontologies by looking for inconsistencies between the taxonomic origin of the annotated gene products, and the implicit taxon specificity of the GO classes. For example, either direct or indirect automated annotation of a bacterial gene product to the class 'lactation' would give a clear indication that either the ontology or the annotation set required some improvement. Although it may seem trivially obvious to a human curator that a bacterial gene product could not be involved in lactation, this connection is not apparent to an automated annotation system. Inclusion of automated checking is essential for detection and correction of flaws in such a system.

Quality control is of critical importance in both the ontology structure and the annotations. To improve both datasets we have developed a system to automatically find inconsistencies between the implicit taxon specificity of GO classes and the species of origin of the annotated gene products. Using this system, inconsistencies are automatically detected and passed on to curators for correction. This work builds upon the prior publication of three logically defined relations (validity, specificity, and relevance) used to link classes in the Gene Ontology with taxonomic classes [8].

## Results

### Specification of taxonomic constraints

The mainstay of this inconsistency detection system is the capture of taxon specificity of GO classes using two new relationships. Where a GO class should only be used for annotation of gene products from a given taxonomic grouping, the relationship used is *only*_*in*_*taxon*. Conversely, where a gene product should never be used for annotation of gene products from a given taxonomic grouping, the relationship is *never*_*in*_*taxon*. The syntax in which this information is recorded, and that of the other associated files, can be viewed at the locations noted in the methods section.

**Figure 1:**
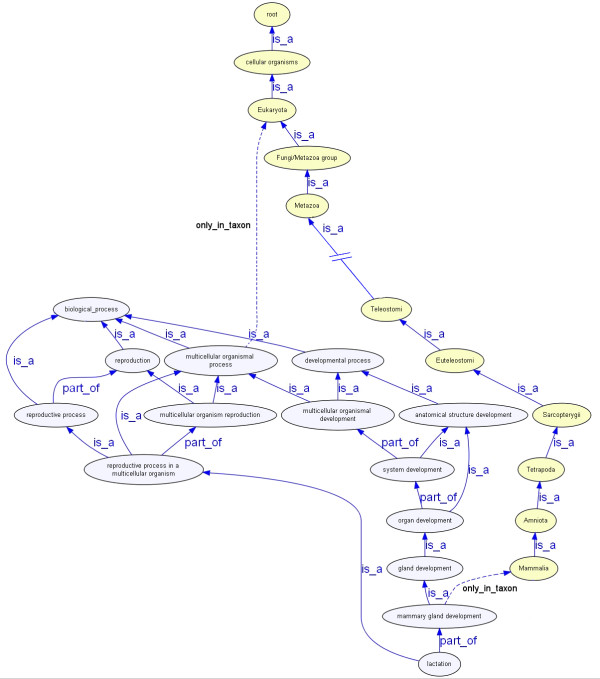
**Lactation**. The GO class 'lactation' is restricted for use with gene products from species of the taxonomic grouping Mammalia. The class inherits this restriction from the superclass 'mammary gland development'. In this figure, the GO classes are shown in blue, and the taxonomic classes are shown in yellow. The relationship types are labeled in the diagram.

Where a GO class X has the *only*_*in*_*taxon *relationship to a taxonomic group Y, this indicates that that GO class and its sub-types and parts should only be used for annotation of gene products from organisms of that taxonomic group and its sub-types. There may be some sub-types of the taxonomic group that do not carry out the process, but there will certainly be no examples of the process outside of the named taxonomic group. To give an example, if the class 'lactation' is restricted to use with Mammalia (lactation *only*_*in*_*taxon *Mammalia - Figure 1), then this class may only be used for annotation of Mammalian gene products. As the relationship is inherited by all Mammalian sub-types, the class can be used for annotation of gene products from species such as *Ornithorhynchus anatinus *(platypus) and *Desmalopex leucopterus *(white-winged flying fox), but not for species outside of Mammalia such as *Arabidopsis thaliana *(thale cress) and *Gallus gallus *(chicken). The constraint is inherited by sub-types and parts of the GO class, and it can be seen in Figure 1 that 'lactation' inherits this constraint from the GO class 'mammary gland development'. The *only*_*in*_*taxon *relationship corresponds to the previously published specificity relationship [8]. The checking system currently contains 443 *only*_*in*_*taxon *constraints (January 2010). We anticipate that there will be scope for a great expansion in the number of constraints, however these are added as the terms are spotted by curators, so the number will continue to build up gradually for some time.

Where a GO class X has the *never*_*in*_*taxon *relationship to a given taxonomic group, this indicates that that GO class and its sub-types and parts should never be used for annotation of gene products from organisms of that taxonomic group or its sub-types. It also indicates that there is no restriction on using the GO class for annotation of gene products from any taxonomic group outside of the one mentioned. To give an example, if the cellular component class 'secretory granule' has the relationship *never*_*in*_*taxon *to the taxonomic group Ascomycota, then that means that the class cannot be used for annotation of gene products from any of the Ascomycota, including *Schizosaccharomyces pombe *(fission yeast) and *Saccharomyces cerevisiae *(baker's yeast). This relationship does not place any restriction on using the class outside of this taxonomic grouping. The *never*_*in*_*taxon *relationship is particularly useful in cases where gene products of some taxa are known to be inappropriate for annotation to a given GO class, but where we do not yet have enough information to make an *only*_*in*_*taxon *grouping, or in situations where it would be inappropriate to make an *only*_*in*_*taxon *relationship because the class is widely applicable, having just a few exceptions. The checking system currently contains only two *never*_*in*_*taxon *constraints, as we try to use the more comprehensive *only*_*in*_*taxon *relationship where possible.

**Figure 2:**
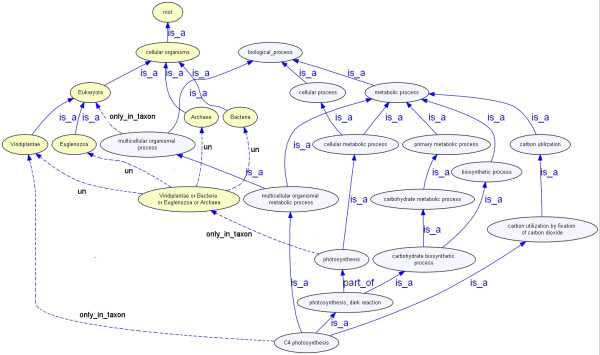
**C4 photosynthesis**. The GO class 'C4 photosynthesis' is restricted for use with gene products from species of the taxonomic grouping Viridiplantae. This is a narrower taxonomic group than that to which the GO superclass 'photosynthesis' is restricted. The GO class 'photosynthesis' is restricted for use with gene products from any sub-type of the Viridiplantae, Euglenozoa, Archaea or Bacteria. The relationship between 'photosynthesis' and these four taxonomic groups is shown by the relationship *only*_*in*_*taxon *from 'photosynthesis' to the union term 'Viridiplantae or Euglenozoa or Archaea or Bacteria', and by the relationships between this union term and the four individual taxonomic groups. These latter relationships are shown as *union*_*of *relationships (marked 'un'). In this figure, the GO classes are shown in blue, and the taxonomic classes are shown in yellow. The relationship types are labeled in the diagram.

Taxon classes are drawn from the NCBI taxonomy hierarchy and supplemented with *union classes *created for use in-house. For example, to capture the set of organisms carrying out photosynthesis in any form we have created the union class 'Bacteria or Archaea or Viridiplantae or Euglenozoa' (Figure 2). This is necessary because sub-types of all of these classes carry out photosynthesis, but in the NCBI taxonomy hierarchy there is no common super-class that includes all of these groups. Where sub-types of a taxon-restricted GO class have narrower implicit taxon specificity than the ancestor class, this is asserted by applying a stricter relationship. For example, photosynthesis is restricted for use with gene products of the group that is the union of 'Bacteria or Archaea or Viridiplantae or Euglenozoa'. However, the sub-type of photosynthesis known in GO as 'PEP carboxykinase C4 photosynthesis' is restricted for use to the smaller Viridiplantae group (Figure 2). This narrower taxonomic group further constrains the applicability of the class relative to the ancestor GO class.

### Consistency checking using taxon constraints

The main utility of this set of formalized constraints is in checking for inconsistencies between the annotations and the ontologies. A script is run once a week to check for annotations that contravene the constraints (see methods). For example, one of the checks is to see if any gene products from species outside of the taxon Mammalia has been annotated to the GO class 'lactation' or to any of its sub-types. Discovery of an annotation contravening such a constraint would give a clear indication that work was required to improve either the ontology or the annotation. All annotations in the GO central repository are checked with each of the constraints, and a set of the inconsistencies flagged is made available to the groups that produced the annotations.

There are several beneficial outcomes of this regular checking. Problems in the ontology structure or annotation set are quickly spotted and corrected. A common type of error is an inaccuracy in the inheritance path down the long series of relationships in the ontology. Though these are hard to spot by eye, they are easy to automatically detect with this new checking system. Another frequently occurring problem is an ambiguity in a GO class definition that may have led annotators to interpret and use classes in a very different way from that intended by the editors. Prompt detection and reporting of such problems greatly enhances the accuracy of the ontology and the speed of correction. One of the most common errors that we have found with the checks is the annotation of a viral gene product to a cellular component term rather than the equivalent 'host' cellular component term. This can particularly be seen with the EXP and TAS annotations (Table 1). In these cases many viral gene products were annotated to terms such as 'endosome lumen' instead of 'host endosome lumen'. As this appears to be a significant issue, we are reviewing our policies on the annotation of viral gene products to these terms. On closer examination we discovered that the majority of these EXP and TAS viral annotations are sourced from Reactome [9] (in fact the only annotations to use the generic EXP code are those sourced from Reactome). We are exploring the possibility of automatically fixing these annotations to use the "host" term.

**Table 1:** Numbers of annotation inconsistencies found, classified by evidence code.

Evidence code type	Evidence code	Annotation errors	Total annotations	Percentage error rate
Experimental (manually assigned)	EXP	977	5360	18.23
	IDA	12	105764	0.01
	IMP	84	88283	0.10
	IEP	0	10129	0.00
	IPI	0	29877	0.00
	IGI	0	12914	0.00

Computational Analysis (manually assigned)	ISS	85	228605	0.04
	ISO	1	2975	0.03
	ISA	0	5921	0.00
	ISM	0	143	0.00
	IGC	0	483	0.00
	RCA	3	75175	0.00

Author statement (manually assigned)	TAS	4070	46888	8.68
	NAS	3	23578	0.01

Curator statement (manually assigned)	IC	0	5682	0.00
	ND	0	171817	0.00

Automatically Assigned	IEA	639	844441	0.08

A large number of inconsistencies have been found, and the problems corrected. The number of inconsistencies in each evidence code group are shown here, both as an absolute number, and as a percentage of the total annotations with that code. Note that the high rate of EXP annotation flags are due to Reactome virus annotations (when this is corrected for, the EXP error rate drops to nearly zero). For interpretation of evidence codes see http://www.geneontology.org/GO.evidence.shtml

The following section shows further specific examples of improvements that have been made to the annotation sets. A summary of the numbers of annotation inconsistencies being flagged by a selection of the constraints is shown in Table 2. It is important to note that inconsistencies may reflect problems in either the annotations or ontologies, even though they are flagged as inconsistent annotations. We have been able to make extensive improvements to both datasets as a result of these checks. A summary of the number of annotation inconsistencies that have been found and fixed is shown in Table 1 sorted by evidence code, and in Table 3 sorted by ontology. These tables do not include annotations from the GOA UniProtKB electronic annotation dataset, as we have not yet been able to fully check this very large dataset. We would like to stress that only a very tiny minority of annotations and GO classes are problematic, reflecting the diligence of GO annotators and ontology developers, and the quality of our electronic annotation methods. This is indicated by the very low percentage error rate shown in the last column of each table. However, even a small number of errors can cause problems for our users, and so we consider this checking system to be a valuable contribution to quality control in the GO dataset.

**Table 2:** Numbers of annotation inconsistencies found by certain rules.

Constraint	Errors detected	Evidence class
GO:0030879	19	IEA
'mammary gland development'		
*only*_*in*_*taxon*		
NCBITaxon:40674		
'Mammalia'		

GO:0012511	21	IEA
'monolayer-surrounded lipid storage body'		
*only*_*in*_*taxon*		
NCBITaxon:33090		
'Viridiplantae'		

GO:0001701	51	IEA
'in utero embryonic development'		
*only*_*in*_*taxon*		
NCBITaxon:32525		
'Theria'		

GO:0001541	10	IEA
'ovarian follicle development'		
*only*_*in*_*taxon*		
NCBITaxon:40674		
'Mammalia'		

GO:0051300	13	Mixture of ISO, ISS, IEA and IMP.
'spindle pole body organization'		
*only*_*in*_*taxon*		
NCBITaxon:4751 'Fungi'		

GO:0015979	9	IEA
'photosynthesis'		
*only*_*in*_*taxon*		
NCBITaxon_Union:0000021		
'Viridiplantae or Bacteria or Euglenozoa or Archaea'		

GO:0015995	9	IEA
'chlorophyll biosynthetic process'		
*only*_*in*_*taxon*		
NCBITaxon_Union:0000007		
'Viridiplantae or Bacteria or Euglenozoa'		

A large number of inconsistencies have been found and various repairs made. This table gives a summary of the numbers of annotation errors found using a selection of the rules that we have implemented. For interpretation of evidence codes see http://www.geneontology.org/GO.evidence.shtml

**Table 3:** Numbers of annotation inconsistencies found, classified by ontology.

Ontology	Annotation errors	Total number of annotations	Percentage errors
Biological process	237	568306	0.04
Molecular function	35	627858	0.01
Cellular component	5602	461910	1.12

A large number of inconsistencies have been found, and the problems corrected. The number of inconsistencies in each ontology are shown here, both as an absolute number, and as a percentage of the total annotations to that ontology.

### Inconsistencies found and fixed -- Electronic annotations

Automated pipelines can quickly produce large volumes of annotation for a diverse set of species. In situations where there is no funded manual annotation program such methods are extremely valuable, but generation methods must be strictly controlled to reduce production of incorrect annotations. A large proportion of the queries returned by this checking system were triggered by automatically generated annotation, and so we conclude that implementation of the system is a valuable contribution to quality control in this area.

As examples of this, *Drosophila *two IEA annotations to GO:0019684 'photosynthesis, light reaction' and eight annotations to GO:0009288 'bacterial-type flagellum' have been caught and removed, prompting a review of the FlyBase automatic annotation pipeline. These spurious annotations arose because of low probability matches between *Drosophila *proteins and short InterPro domains. The automated Interpro2GO pipeline mapped these false positive domain hits to GO classes. By increasing the stringency for InterPro domain to protein mapping, these taxon errors have been eliminated and the confidence level of all IEA-based GO assignments has improved in FlyBase.

Similarly, automatic transfers of annotations to orthologs needed to be further restricted when the class GO:0001701 'in utero embryonic development' was found to have been transferred from a mammalian gene product to an avian gene product by Ensembl Compara [3] for 144 annotations.

### Inconsistencies found and fixed -- Manual annotation or ontology development

Excluding the viral EXP annotations, the majority (77%) of remaining inconsistencies found were derived from unvetted automated prediction programs, but errors were also found in experimentally derived and manually checked annotations. Some problems in manual annotations were found to have resulted from misunderstandings of the meanings of GO classes between the ontology editors who wrote the class definitions and the annotators who were using them. For example, the class 'sensory perception' was originally defined as 'The series of events required for an organism to receive a sensory stimulus, convert it to a molecular signal, and recognize and characterize the signal.'. To an annotator reading the class name and definition it would seem that this class could be used for annotation of bacterial gene products that enable the bacterium to sense and recognize outside influences. However, the GO class has in its ancestry the class 'cognition', indicating that this is a neurological process and therefore not suitable for annotation of bacterial gene products. To avoid future annotation errors, the definition was clarified by the addition of the sentence: 'This is a neurological process.'. The incorrect bacterial annotations were removed from the source database.

In some cases the class names and definitions can be quite subtle and gene products can accidentally be annotated to classes that are almost, but not quite correct. For example the fungal microtubule organizing center is called the 'spindle pole body', whilst in mammals the microtubule organizing center is called the 'centrosome'. In GO we have classes for 'centrosome organization' and for 'spindle pole body organization', and only fungal gene products should be annotated to the 'spindle pole body organization' class.

Application of a taxon constraint has enabled annotations applied to this class in error to be caught and corrected. Having caught this kind of error once, the ontology developers can improve the definition so that in future the meaning will be more apparent to annotators. This kind of check is particularly useful where a constraint has been applied to a fairly high-level class, showing up ambiguity and consequent errors in the use of the sub-types of the class. The advantage here is that all the sub-types do not need to be individually considered for application of constraints, but that they can be caught using a single high-level constraint.

A small number of other inconsistencies were found to have been brought about by typing errors in accession numbers, and these have been fixed.

### Novel electronic annotations

In addition to preventing errors, the new system enables us to produce a large volume of new electronic annotations. In previous years many mappings have been omitted from the InterPro2GO mapping files, because they would not be applicable to all species. However, now such mappings can be used in conjunction with the taxon constraints to ensure that annotations are only transferred to gene products from appropriate species. The new combined system will enable generation of a very large body of novel electronic annotation.

## Discussion

In creating this system we have examined the previously published relationship options [8] and adapted them to provide a simple, useful checking system. This has brought about improvement to both the annotation set and the ontologies.

In developing the taxon constraints there is always the concern of over- or under-constraint. If the constraints are too tight then we risk flagging correct annotations, whilst if the constraints are too loose, we risk failing to detect problems in the annotations or ontologies. The system has been designed to work hand-in-glove with the manual annotation and ontology development processes, so that there is a virtuous circle of error detection and correction. To best integrate the system into our existing processes, we have chosen to start with excessively tight constraints, and then immediately correct any errors in the constraints that are shown up by the annotation set. As we have a very large and diverse annotation set available, errors in the constraints can be quickly detected and removed.

Currently we have not integrated the taxonomic constraints directly into automated function prediction tools, instead opting to use the constraints to vet the resulting annotations, to minimize false positives during error detection. We are currently integrating the constraints into our Phylogenetic Annotation INference Tool (PAINT) [10], which allows a curator to rapidly propagate experimental annotations across species using common descent in a semi-automated fashion. The curator is notified if an attempt to propagate an annotation violates a constraint.

In addition to the core utility of annotation and ontology checking, the development of this system brought about a few other interesting avenues for exploration. The most notable surprise to us was the frequency with which we found annotations indicating horizontal gene transfer between viral and host genomes. For example, one check flagged a viral gene product that encodes a component of the photosynthetic machinery [11]. Another set of checks highlighted functional gene products encoded by an endogenous retrovirus in the mouse that can produce mature envelope proteins [12]. Clearly this information was already in the scientific literature for individual gene products, however our system has fortuitously shown us a way to automatically mine such cases. This situation presents a slight difficulty for the checking system, as viral gene products are being found in many processes that would be expected to be carried out only by the host. To accommodate this we are keeping the checks that flag these cases, but then the database groups are ignoring the flagged gene products in the violations file. We may investigate more satisfactory approaches in the future - for example, indicating genes that arose through horizontal transfer in the annotations.

Creation of the union classes also gave us an opportunity to reflect on the diversity of taxonomic groups that carry out very similar processes. Initially we created a union class 'Viridiplantae or Bacteria' as a constraint for the high level class 'photosynthesis'. Annotation checking showed us that we needed to expand this to 'Bacteria or Archaea or Viridiplantae or Euglenozoa'. This demonstrated to us an interesting automated method for detection of diverse taxonomic groups that carry out very similar processes. It should be noted that the union classes do not give any indication of the relatedness of taxonomic groups, or of either convergent or divergent evolution in the past. They simply give an indication of which diverse groups of organisms might be investigated for either phenomenon.

## Conclusions

The GO Consortium provides a highly developed ontology structure associated with a large volume of annotations. It is essential that a range of automatic checks are carried out on these resources to ensure provision of a maximally correct dataset. The feedback generated from the described taxon checking system has benefited both ontology development and annotation in the GO Consortium. It is intended that the simple format of these taxon sanity checks will allow GO annotation providers and external GO annotation prediction tools to directly integrate the checks into their tools, so that annotations can, in future, be checked pre-release. Such efforts are already being undertaken by the UniProtKB-GOA and InterPro groups at the EBI.

## Methods

### Generation of taxon-slim

We converted the NCBI taxonomy from a tab delimited format to an ontology in OBO format using a custom translation that preserves information such as synonyms and taxonomic rank. The basic relation used is *isA*. The file is available from the OBO registry [13].

The full taxonomy contains 357849 classes (February 2010) and is difficult to browse due to the depth at which species taxa reside. In practice we only need a subset for the human-guided selection of constraints. We used a custom implementation of the MIREOT method [14], taking all leaf nodes (species) with human curation in the GO, and generating a slim version of the taxonomy. The slim includes only those taxa that are annotated species nodes, or the least-common-ancestor of any two species nodes.

Although the taxon constraints are created using an NCBI slim, the checks use the entire NCBI taxonomy hierarchy, ensuring that any new species added to an annotation set will be checked. The NCBI taxonomy slim is useful for editing, and can just be periodically updated. However, it is important to carry out the inconsistency checks using the entire NCBI taxonomy, as the UniProt set of annotations expands to cover around 4,500 new species with every data release. New species could easily fail to inherit constraints if only the slim was used. For example, in the case of the class lactation, in any given month, annotations may be made with gene products from a type of mammal species like *Lasiurus seminolus *(the Seminole bat) that was previously not included in the in-house NCBI slim. When this occurs, use of the full NCBI taxonomy for checking ensures that that species will still correctly inherit any taxon constraints, and that it will be correctly checked by the script.

### Automated support for generation and selection of constraints

Generation of the GO taxon constraints was primarily a manual process, using biological knowledge. We also seeded the constraint checks by querying the GO database using the AmiGO GOOSE interface [15] for classes that lacked annotations within a given taxon, but that had annotations in sister taxa. This method is not fully reliable due to under-annotation. For example, there may be no annotations from species other than *Arabidopsis thaliana *to the class 'stipule development', but this merely reflects a lack of experimental evidence, or a lack of annotation. It does not indicate that stipule development is absent from all other species. We manually vetted all the constraints that were seeded in this way.

We also used the deprecated 'sensu' designators in GO to seed some of the taxon constraints. This resulted in an over-constrained set, so the set was manually vetted [2]. The sensu designations previously captured a form of taxonomic information, but were commonly misunderstood by users, and therefore have recently been removed and replaced by more comprehensive class definitions and clearer class names. For example, there was previously a class called 'eye development (sensu Insecta)', where the sensu designation indicated 'eye development as found in the taxonomic group Insecta'. This class is now called 'compound eye development', and it has a comprehensive definition that does not require taxonomic context for clarity. Although the sensu designations have now been removed, the historical use of a sensu designation gives a valuable clue to where implicit taxon specificity might be found.

### Detection of constraint violations

The constraint system is implemented using the GOBO perl toolkit [16]. GOBO includes a backward-chaining inference engine, which is used to calculate the link between any given class in the GO and a taxonomic group. In addition, there is a reference implementation written in SWI-Prolog [17] that uses the biological logic programming toolkit [18].

Relation composition rules were derived from the formal definitions provided in the OBO Relations Ontology [19]. These are:

$$\begin{array}{c} \text{is}\_\text{a}\left(A,B\right),\text{ is}\_\text{a}\left(B,C\right)\to \text{is}\_\text{a}\left(A,C\right)\\ \text{is}\_\text{a}\left(A,B\right),\text{ part}\_\text{of}\left(B,C\right)\to \text{part}\_\text{of}\left(A,C\right)\\ \text{part}\_\text{of}\left(A,B\right),\text{ is}\_\text{a}\left(B,C\right)\to \text{part}\_\text{of}\left(A,C\right)\\ \text{is}\_\text{a}\left(A,B\right),\text{ occurs}\_\text{in}\left(B,C\right)\to \text{occurs}\_\text{in}\left(A,C\right)\\ \text{occurs}\_\text{in}\left(A,B\right),\text{ is}\_\text{a}\left(B,C\right)\to \text{occurs}\_\text{in}\left(A,C\right)\\ \text{is}\_\text{a}\left(A,B\right),\;U=B\vee C\to \text{is}\_\text{a}\left(A,U\right)\\ \text{annotated}\left(G,A\right),\;(\text{is}\_\text{a}\left(A,B\right)\vee \text{part}\_\text{of}\left(A,B\right)\vee \text{occurs}\_\text{in}\left(A,B\right))\;\to \text{ annotated}\left(G,B\right)\\ \text{in}\_\text{taxon}\left(G,T\right),\text{ is\_a}(T,T’)\to \text{in}\_\text{taxon}(G,T’)\\ \text{never}\_\text{in}\_\text{taxon}\left(A,T\right),\text{ in}\_\text{taxon}\left(G,T\right)\to \neg \text{annotated}\left(G,A\right)\\ \text{only}\_\text{in}\_\text{taxon}\left(A,T\right),\;\neg \text{in}\_\text{taxon}\left(G,T\right)\to \neg \text{annotated}\left(G,A\right) \end{array}$$

Note that the regulates relations are not used here. This is because it is possible to regulate processes in other species. The additional occurs_in relation is specified in an experimental extension of the Gene Ontology [20]. For example, constraints for the class *nucleus *are inherited by the class *nuclear translation*, because this process occurs in the nucleus.

### File availability

All files in this system are publicly available from GO cvs at: go/quality_control/annotation checks/taxon_checks/ and in the online GO cvs browser at: http://www.geneontology.org/quality_control/annotation_checks/taxon_checks/ 

The files are named as follows:

• ncbi_taxon_slim.obo - The NCBI taxonomy slim that we use in-house for editing purposes.

• taxon_go_triggers.obo - The list of GO classes and taxonomic groupings, with the relationships between them shown as *only*_*in*_*taxon and never*_*in*_*taxon*.

• taxon_violations.txt - The file listing annotations that show inconsistencies.

• taxon_union_terms.obo - The le of in-house taxonomic groupings that are unions of two or more NCBI taxonomy groupings.

• taxon_union_materialized_terms.obo - As the previous le, but with the is_alinks materialized from the union definitions, as define above.

• taxon_go_imports.obo - The file that can be called by the ontology editor tool OBO-Edit [21] to automatically load all of the other files for editing or browsing.

### Numbers of inconsistencies found

Several tables are provided to give in an indication of the numbers of inconsistencies found with our initial set of constraints. Table 2 was made using annotation and constraint files from February 2010. The figures in Table 1, and Table 3 were generated using the inconsistency file of 23rd March 2010 with the annotation files from 1st January 2010. The older annotation set was used with the newer constraint file as we had spent time in between correcting the annotation set and the taxonomic constraints file. To give a clear idea of the efficacy of the checking system it made sense to gather data using the older uncorrected annotation files with the newer corrected inconsistency checking file (from which overly strict constraints had been removed). The GOA UniProtKB IEA dataset is not included in these tables, as the dataset is so large that we have not yet been able to process it.

## Authors' contributions

JID and CJM jointly designed the system, developing new relationship types appropriate for capture of this taxon specificity information. JID implemented the system, collecting taxon constraints and devising taxon union classes. She did this under the supervision of CJM, who developed the computational aspects of the implementation. ECD extensively vetted the output of the inconsistency checks and spotted interesting trends. She also fed back valuable information on how the system could be improved. All authors read and approved the final manuscript.
